# The effect of single low-dose primaquine treatment for uncomplicated Plasmodium falciparum malaria on hemoglobin levels in Ethiopia: a longitudinal cohort study

**DOI:** 10.21203/rs.3.rs-4095915/v1

**Published:** 2024-03-15

**Authors:** Kassahun Habtamu, Hallelujah Getachew, Ashenafi Abossie, Assalif Demissew, Arega Tsegaye, Teshome Degefa, Xiaoming Wang, Ming-Chieh Lee, Guofa Zhou, Solomon Kibret, Christopher L. King, James W. Kazura, Beyene Petros, Delenasaw Yewhalaw, Guiyun Yan

**Affiliations:** Addis Ababa University; Arbaminch College of Health Sciences; Arba Minch University; Ambo University; Jimma University; Jimma University; University of California at Irvine; University of California at Irvine; University of California at Irvine; West Valley Mosquito and Vector Control District; Case Western Reserve University; Jimma University; Addis Ababa University; Jimma University; University of California at Irvine

**Keywords:** P. falciparum malaria elimination, Primaquine, Artemisinin-based combination therapies, Hemoglobin, G6PD deficiency, Ethiopia

## Abstract

**Background:**

To interrupt residual malaria transmission and achieve successful elimination of *P. falciparum* in low-transmission settings, the World Health Organization (WHO) recommends the administration of a single dose of 0.25 mg/kg (or 15 mg/kg for adults) primaquine (PQ) combined with artemisinin-based combination therapy (ACT) without glucose-6-phosphate dehydrogenase (G6PD) testing. However, due to the risk of hemolysis in patients with G6PD deficiency (G6PDd), PQ use is not as common. Thus, this study aimed to assess the safety of a single low dose of PQ administered to patients with G6PD deficiency.

**Methods:**

An observational cohort study was conducted with patients treated for uncomplicated *P. falciparum* malaria with either single-dose PQ (0.25 mg/kg) (SLD PQ) + ACT or ACT alone. Microscopy-confirmed uncomplicated *P. falciparum* malaria patients visiting public health facilities in Arjo Didessa, Southwest Ethiopia, were enrolled in the study from September 2019 to November 2022. Patients with uncomplicated *P. falciparum* malaria were followed up for 28 days through clinical and laboratory diagnosis, such as measurements of G6PD levels and hemoglobin (Hb) concentrations. G6PD levels were masured by a quantiative biosensor machine. Patient interviews were also conducted, and the type and frequency of clinical complaints were recorded. Hb data were taken on days (D) 7, 14, 21, and 28 following treatment with SLD-PQ + ACT or ACT alone.

**Results:**

A total of 249 patients with uncomplicated *P. falciparum* malaria were enrolled in this study. Of these, 83 (33.3%) patients received ACT alone, and 166 (66.7%) received ACT combined with SLD-PQ treatment. The median age of the patients was 20 (IQR 14) years. G6PD deficiency was found in 17 (6.8%) patients, 14 males and 3 females. There were 6 (7.2%) and 11 (6.6%) phenotypic G6PD-deficient patients in the ACT alone and ACT + SLD-PQ arms, respectively. The mean Hb levels in patients treated with ACT + SLD-PQ were reduced by an average of 0.45 g/dl (95% CI = 0.39 to 0.52) in the posttreatment phase (D7) compared to a reduction of 0.30 g/dl (95% CI = 0.14 to −0.47) in patients treated with ACT alone (*P* = 0.157). A greater mean Hb reduction was observed on day 7 in the G6PD deficiency group (−0.56 g/dL) than in the G6PD normal group (−0.39 g/dL); however, there was no statistically significant difference (*P* = 0.359). Overall, D14 losses were 0.10 g/dl (95% CI = −0.00 to 0.20) and 0.05 g/dl (95% CI = −0.123 to 0.22) in patients with and without SLD-PQ, respectively (*P* = 0.412).

**Conclusions:**

Our findings showed that single low-dose primaquine (SLD-PQ) treatment for uncomplicated *P. falciparum* malaria is safe and does not increase the risk of hemolysis in G6PDd patients. This evidence suggests that the wider deployment of SLD-PQ for *P. falciparum* is part of a global strategy for eliminating *P. falciparum* malaria.

## Background

The global decline in malaria incidence due to increased malaria control measures, particularly the use of artemisinin-based combination therapy (ACT), has stimulated efforts to eliminate *Plasmodium falciparum* and sparked an upsurge in interest in therapies to stop transmission [1]. In many malaria-endemic areas, artemisinin derivatives are very effective against both asexual and young *P. falciparum* gametocytes, and this has considerably enhanced the global reduction in malaria transmission[2]. However, mature gametocytes continue to exist after ACT at the microscopic or submicroscopic level, and residual transmission is not interrupted after ACT[3, 4]. This is the reason that artemisinin derivatives against mature *P. falciparum* gametocytes are not effective in preventing transmission[5].

To treat *P. falciparum* malaria, the World Health Organization (WHO) recommends incorporating a single low dose of primaquine (SLD-PQ, 0.25 mg/kg of body weight) into artemisinin combination therapy (ACT) as part of preelimination or elimination programs[6] and as part of artemisinin resistance containment programs[7]. In low-endemic settings, the combination of this gametocytocidal drug with ACT is effective[8], and this combination may significantly reduce malaria transmission [9]. Furthermore, there is strong evidence that primaquine can prevent relapses of *P. ovale* and *P. vivax*[10]. The WHO currently recommends adding a single dose of 0.25 mg/kg to ACTs without glucose-6-phosphate dehydrogenase (G6PD) to reduce transmission in low-transmission settings and accelerate the elimination of *P. falciparum*[8, 11]. However, due to fears of hemolysis in people with G6PD deficiency (G6PDd) [12], the use of PQ is not as common as anticipated. Hence, the broad use of PQ is hampered by safety concerns of hemolysis in individuals with G6PDd, the most prevalent hereditary enzyme defect reported in all malaria-endemic areas[13].

More than 400 million people worldwide suffer from G6PDd, which is an X-linked genetic characteristic with an average frequency of 8% (range 3 to 30%) in malaria-endemic countries[14]. The frequency of G6PDd varies significantly from region to region, even within a country, with the highest prevalence reported in Africa. Additionally, migration and resettlement have an impact on the spread of this disease[15]. Due to safety concerns, especially for those with G6PDd, which affects up to 37.5% of the continent’s population[16], the use of PQ is also restricted, particularly in Africa. Therefore, worries about the safety of PQ in relation to hemolysis at the individual level have prevented its use despite the benefit being solely at the population level[17].

Although evidence on the safety of the administration of SLD-PQ has increased, there is indirect evidence that this SLD-PQ is well tolerated in G6PDd patients. Several randomized and controlled clinical trials on the safety and efficacy of ACT versus SLD-PQ have been conducted in South Africa[17], Tanzania[18, 19], Senegal[20], Switzerland[21], Burkina Faso[22], and Uganda[23]^,^ supporting the use of the WHO-recommended SLD-PQ without G6PD testing during the preelimination and elimination phases of malaria.

Although previous studies have shown that SLD-PQ is safe even for people with G6PDd, the WHO recommended additional clinical research to ensure the safety of SLD-PQ in G6PDd individuals in different eco-epidemiological settings[24]. Furthermore, due to pragmatic dosing techniques, some patients may receive a dose greater than the recommended dose of 0.25 mg/kg, which may improve gametocyte clearance but increase the risk of hemolysis [25].

Unfortunately, most studies were clinical trials that lacked population-based data and merely provided quantitative data assessing the effect of the SLD-PQ on hemoglobin (Hb) levels. In addition, previous safety studies were often based on carefully chosen study participants and small groups with relatively high pretreatment Hb levels; therefore, they offered limited information at the population level. The risk of anemia is likely to increase in individuals with lower pretreatment Hb concentrations[26]. Although PQ is linked to a brief reduction in Hb levels in G6PDd patients, baseline Hb levels continue to be the leading cause of anemia in such patients[27]. Additionally, severe hemolytic events might be rare and unlikely to be observed in small safety studies[22]. Therefore, whether certain groups are still at risk of clinically significant hemolysis when SLD-PQ treatment occurs at the population level needs to be examined. For noncurative treatments that aim to help the community rather than just the dosed individual, this safety profile is particularly crucial[28]. To address this issue, we evaluated the safety of adding a single fixed low dose of PQ (15 mg tablet or 0.25 mg/kg for a person weighing 60 kg) to ACT regimens of artemether-lumefantrine (AL) to treat patients with *P. falciparum* malaria in an Ethiopian cohort.

This observational cohort study has not yet been carried out in Ethiopia, and owing to the unique epidemiology of malaria, patients with confirmed enzymatic G6PDd were included in an effort to more accurately reflect real-world circumstances. Moreover, changes in the Hb concentration over time were also examined, which is helpful information for clinical practice. Furthermore, previous studies have mainly examined one population group—males[22], children[29], adults[30], or asymptomatic people[31]—while excluding patients with G6PDd[3]. In the present study, all groups were included.

## Methods and materials

### Study Area

This study was conducted at the Arjo-Didessa SugarCane Plantation and surrounding areas of southwestern Ethiopia between September 2018 and November 2022. Arjo-Didessa is located 540 km southwest of Addis Ababa, the nation’s capital. The latitude of the study area is 8°41′35.5″N, and the longitude is 36°25′54.9″E. The mean annual rainfall is 1477 mm, which falls over two rainy seasons: one short season between February and March and the other long rainy season ranging between June and September. These rainy seasons correspond to low and high peak transmission seasons, respectively. The two primary malaria parasite species are *P. falciparum* and *P. vivax*[32, 33]. Studies in this area have reported *P. ovale* infections[34]. The rates of malaria transmission are low, unstable, and seasonal. The vast majority of people living in the area are farmers who raise food crops, including corn, sorghum, nuts, and peppers, and a smaller portion of these people work at the Arjo sugar factory and sugarcane plantations. The residents also maintained livestock to help with their living. The study was carried out in health facilities located in three districts: Dabo Hana (Kerka Health Post and Sefera Tabiya Health Center), Jimma Arjo (Arjo-Dedesa Sugar Factory Clinic, Abote Didessa Health Center), and Bedele (Command 2 and Command 5 Health Posts). The selection of health facilities was based partly on how close they were to the follow-up study.

### Study Design

This was a prospective cohort study on the impact of a single low dose of primaquine on the prevention and control of *P. falciparum* malaria. As part of this study, patients treated for uncomplicated *P. falciparum* malaria with either ACT + SLD-PQ at 0.25 mg/kg or ACT alone were enrolled and assessed for the safety of the addition of SLDPQ. Then, clinical and laboratory evaluations were conducted on days (D) 0, 7, 14, 21, and 28 or on any day of recurrent illness[35]. Patients who satisfied the study’s eligibility criteria, such as uncomplicated *P. falciparum* mono-infection verified microscopically, provided consent to adhere to the study protocol and were included in the study.

The study excluded pregnant women, lactating mothers, and children under one year old due to contraindications to PQ in these populations[36]. Any history of severe malaria or warning signs, a recent history of blood transfusion, febrile conditions unrelated to malaria, or a known chronic or serious underlying illness were among the exclusion criteria.

### Sample size calculation

This study took the primary objective—the mean Hb reduction—into consideration when calculating the proper sample size. The Hb reduction calculation in this study was based on a study by Senegalian, which showed that adding primaquine (0·25 mg/kg) to ACT reduced the mean Hb concentration from 13.4 to 11.9 g/dl (1.47)[6]. With a 10% loss to follow-up and a clinically meaningful noninferiority margin of 0.3 g/dl, the intended sample size for the effect of SLD-PQ on Hb was 131 participants per group. This made it possible to determine if the study group and the reference group were not significantly different by 80% at the two-tailed 5% significance level.

### Sampling procedure

Following the detection of *P. falciparum* on D0, artemether + lumefantrine (AL) tablets twice daily for three days (Coartem [20 mg of artemether and 120 mg of lumefantrine]; Ipca Laboratories Ltd., India) were given for all positive uncomplicated *P. falciparum* patient malaria cases as per the National Malaria Treatment Guidelines [37, 38]. In addition, SLD-PQ + ACT was also administered on D0 in selected health clinics and posts in the study area.

### Laboratory procedure

Blood samples for malaria diagnosis using microscopy and dried blood spots for PCR analysis were taken prior to treatment. In addition, capillary blood samples (300 microns) were collected for Hb concentration measurement and determination of G6PD enzyme activity. On the initial treatment day (D0), the patient was followed up via scheduled appointments on D7, D14, D21, and D28 for Hb status and microscopy examination. Clinical data, including patient sociodemographic information, malaria symptoms, and history of treatment with antimalarial drugs, were collected during enrollment.

### Microscopy of Malaria parasites

According to the WHO malaria microscopy procedure[39], thick and thin smears were taken at patient enrollment and during follow-up, and they were quickly stained with 10% Giemsa. Two different microscopists examined each microscope slide. When there was disagreement about the presence of parasitemia or whether there was a difference in parasite density of more than 25%, a third independent reading was performed[40]. Accordingly, microscopic examination was carried out on D 0, 7, 14, 21, and 28 using Giemsa-stained blood films at a magnification of 1000x.

### G6PD enzyme and Hb measurements

Blood samples from each study participant were tested for G6PD enzyme and Hb levels in accordance with the manufacturer’s instructions. We used CareSTART^™^ POCT S1 (Access Bio, Inc., New Jersey, USA) to determine the Hb and G6PD levels. As recommended by the manufacturer, Hb and G6PD activity were checked daily prior to sample collection. Every sample was run twice. International units per deciliter, or IU/dl, are used to express G6PD enzyme activity, which was measured using a G6PD strip. The enzyme activity was normalized using Hb values taken concurrently with the G6PD enzyme test and expressed as IU/g Hb. Quantitative POCTs were performed using “biosensors,” portable electronic devices where disposable test strips were inserted. Within five minutes after blood was added, the device’s screen displayed the results as a quantitative readout. An unused test strip was inserted into the biosensor, and an aliquot from each blood sample (5 μL) was then placed on the exposed end of the test strip until the device displayed complete automated sample intake. The running times for the G6PD and Hb tests were 4 min and 10 sec, respectively. Using a previously described methodology[41], the adjusted male median (AMM) G6PD activity (100% G6PD activity) for males was used to determine the cutoff values for G6PD deficiency. After excluding samples with activity levels less than 10% of the overall median, the AMM was determined to be the median G6PD activity of all male participants. For male study participants, class I was G6PD deficient with less than 30% AMM activity, while class II was normal with more than 30% AMM activity. Individuals with G6PD activity < 30%, 30–80%, or > 80% of the AMM activity in females are regarded as G6PD deficient, intermediate, or normal, respectively[42–45].

### Clinical and safety assessments

Safety assessments in the present study were performed via hematological analysis and recording of all adverse events[46]. All patients were asked without probing about their health after taking their last dose of antimalarial medication, and the responses were categorized as none, mild, moderate, severe, or life-threatening. The following adverse events (AEs) were recorded: abdominal pain, nausea, vomiting, passing through dark urine, itching, rash, and any drug-related severe adverse events (SAEs). AEs were graded using the Primaquine Roll Out Monitoring Pharmacovigilance Tool (PROMPT), a tool that is helpful for assessing the safety of AEs and SAEs[21]. Mild, moderate, severe, and life-threatening AEs were further divided into four categories: mild, easily tolerable; no or little interference with daily activities; moderate, low level of inconvenience; more than little interference with daily activities; severe, interrupted regular daily activities, typically incapacitating; and life-threatening, life-threatening consequences, indicating the need for immediate intervention or death events.

### Outcomes

The primary outcome of this study was the mean Hb level of the patients following the addition of SLD-PQ in combination with ACT between days 0 and 7[47]. The changes in mean Hb concentrations were taken at baseline values (day 0) in grams per deciliter to compare with (i) mean Hb reduction on D7; (ii) mean Hb variation over the follow-up period; and (iii) Hb recovery (D28 Hb > D0 Hb concentration). The secondary outcomes of this study were the occurrence of adverse events (AEs), including blood transfusions, AEs that occurred within 7 and 28 days after the administration of primaquine, and other AEs related to the administration of PQ, such as gastrointestinal reactions within 7 days and a risk of hemolysis.

### Data management and analysis

The data were entered into a Microsoft Excel data sheet and analyzed using Stata (v17, StataCorp LLC, Texas). A t test was used to determine the mean difference at baseline and on days 8, 15, 22, and 29 following the SLD-PQ in each group. The mean changes in Hb concentration between patients in the ACT alone and ACT + SLD-PQ arms were assessed using an independent sample t test. A linear mixed model with repeated measurements was used to examine mean Hb changes from baseline. To assess whether there was a significant difference in the mean Hb change between G6PD-deficient patients and normal patients following therapy, we employed a multiple linear regression model adjusted for age, sex, and baseline Hb concentration. The reported complaints and adverse events (AEs) in the ACT alone and ACT + SLD-PQ treatment arms were also compared using Fisher’s exact test.

## Results

### Overview of the study

**Figure 1:**
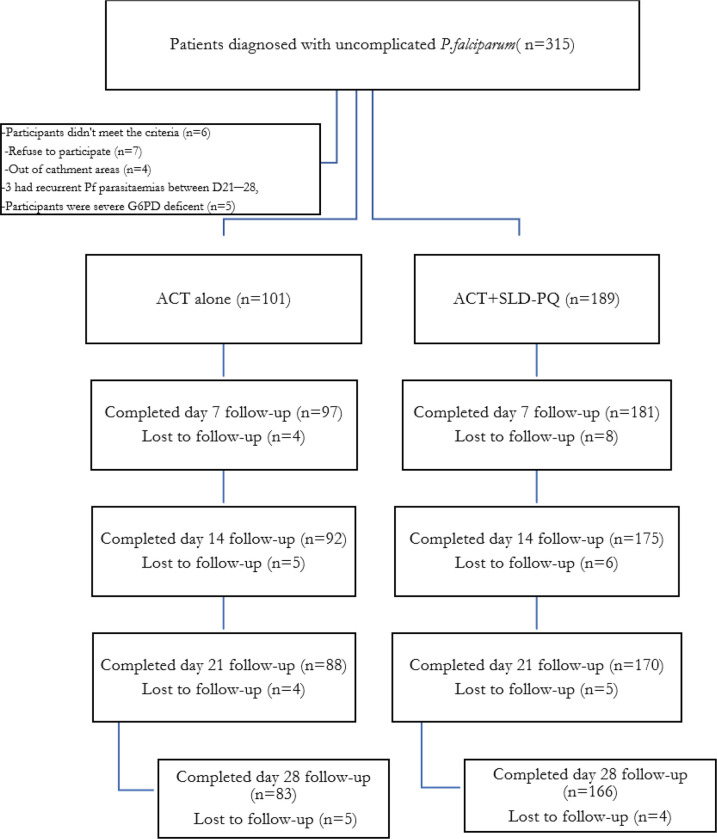
Flow chart of uncomplicated *P. falaciparum* malaria patients enrolled for ACT and SLD PQ + ACT in Arjo Didessa, Southwest Region, Ethiopia ACT, artemisinincombination therapy; PQ, primaquine; *P. falciparum*, *Plasmodium falciparum.*

A total of 315 febrile patients with confirmed *P. falciparum* malaria were followed for two seasons during high malaria transmission. The study excluded 66 individuals who were not within the catchment area, did not match the inclusion criteria, had severe G6PDd, or declined to participate (Figure 1). The remaining 249 (79%) patients were eligible and completed the 28-day follow-up. The initial characteristics of the two treatment groups were comparable (Table 1). Patient sociodemographic characteristics included the total number of patients who received ACT alone (n = 83) or ACT+SLD-PQ (n = 166) for uncomplicated *P. falciparum* malaria infection. Adults (aged > 15 years) accounted for the majority of the study population, with a total of 197 (79.12%) individuals and a sex ratio of males to females of 1.8 (Table 1).

### Phenotypic G6PD status

**Figure 2:**
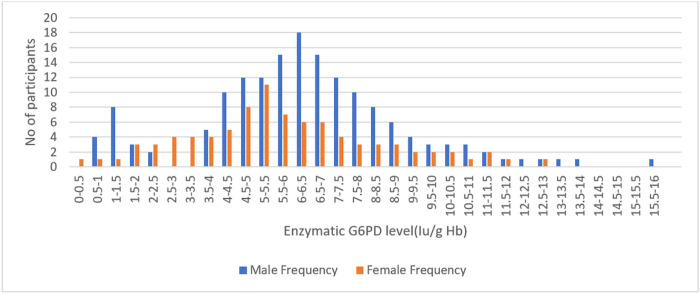
Distribution of G6PD enzyme levels by sex at Arjo-Didessa, southern Ethiopia; 2019–2022.

At the time of enrollment, phenotypic point-of-care testing was utilized to screen patients for G6PD deficiency. The adjusted median G6PD enzyme activity was determined for the male participants by excluding 5 patients whose enzyme activity was less than 10% (less than 0.627 U/g Hb) of the median value found for all male participants. The median G6PD activity was 6.32 U/g Hb for all study participants (the range was 0.4–15.22 U/g Hb). The AMM (range) enzyme activity was 6.32 IU/gHb (0.67–15.219), which was 100%. The distribution of enzymatic activity by sex is illustrated in Figure 2. There was a distinct bimodal distribution in males and a unimodal distribution in females. When the participants’ enzyme activity was between 0.4 and 1.896 U/g Hb (less than 30% of the adjusted male median), they were classified as deficient. Phenotypic G6PD data were recorded for 249 patients; 17 (6.83%) had G6PDd—14 males and 3 females. In the ACT alone arm, there were 6 (2.41%) patients with phenotypic G6PDd, whereas there were 11 (4.42%) patients in the ACT + SLD-PQ arm (Table 1).

### Hemoglobin profiling

**Figure 3:**
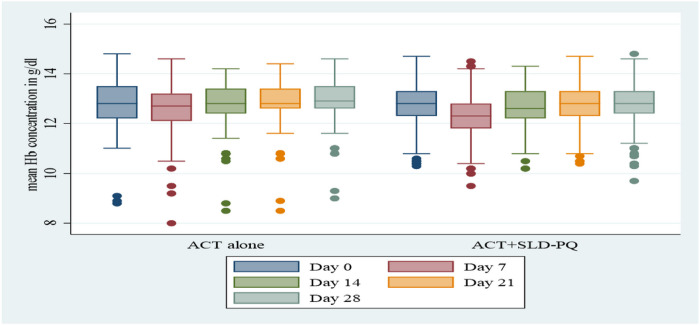
Mean hemoglobin levels (± SE) during the 28-day follow-up (D0, D7, D14, D21, and D28) in malaria patients treated with ACT alone and SLD-PQ +ACT Key: ACT,artemisinin combination therapy; Hb, hemoglobin; SLD-PQ, single low-dose primaquine. D0, day of enrollment; D7, day 7 after treatment; D14, day 14 after treatment; D21, day 21 after treatment; D28, day 28 after treatment

Data from 415 Hb measurements in 83 patients treated with ACT alone and 830 Hb measurements in 166 patients treated with ACT+SLD-PQ were used for the assessment of the changes in the mean Hb level between baseline and days 7, 14, 21, and 28. During enrollment, the mean (SD) Hb level was 12.80 (1.18) g/dL for patients receiving ACT alone and 12.75 (0.89) g/dL for those receiving ACT+SLD-PQ (Table 1). There was no significant difference in the baseline Hb levels between the two treatment arms (*p* = 0.7357). However, males had greater mean (SD) Hb concentrations at baseline (12.78 (1.03) [95% CI 12.62, 12.94]) than females did (12.75 (0.93) [95% CI 12.55–12.94]). There were no significant differences in the mean Hb concentration between males and females in the two treatment arms (*p* = 0.8281). However, as illustrated in Figure 3, in both groups receiving ACT+SLD-PQ, the mean Hb concentrations, shown as the mean change, decreased in the first week following treatment and reverted to baseline values during follow-up. Similarly, paired analysis of Hb concentrations relative to baseline demonstrated a reduction in the first week after receiving 0.25 mg/kg PQ + ACT or ACT alone. These reductions were significant on day 7 following SLD-PQ (0.25 mg/kg PQ) (*P* < 0.01) or ACT alone (*P* < 0.01; Table 2). However, there was no significant difference between the treatment groups, ACT alone and ACT+SLD-PQ (*P* = 0.157; Table 3).

After posttreatment (D7), Hb levels decreased on average by 0.45 g/dl (95% CI =0.39 to 0.52) in patients receiving ACT +SLD-PQ compared to 0.30 g/dl (95% CI =0.14 to 0.47) in patients receiving ACT alone (*P* = 0.157; Table 3). Patients with and without SLD-PQ experienced overall (D0–14) Hb losses of 0.10 g/dl (95% CI = −0.00 to 0.20) and 0.05 g/dl (95% CI = −0.123 to 0.22), respectively (*P* = 0.412; Table 3). At the overall follow-up (D0–28), the mean Hb levels were marginally lower in patients treated with ACT+SLD-PQ than in those not treated with ACT+SLD-PQ (Figure 3). Patients in the ACT alone and ACT + SLD-PQ arms recovered their Hb to or above the baseline values by day 28 (Figure 3).

### Mean Hb (SD) changes following treatment

The mean reductions in Hb were [−0.05 ( 0.79)] g/dl and [−0.10 (0.68)] g/dl in the ACT alone and ACT + SLD-PQ arms, respectively, on day 14. The mean reductions in Hb were 0.04 (0.90) g/dl and 0.04 (0.61) g/dl for the ACT alone and ACT + SLD-PQ arms, respectively, on day 21. The day 28 mean reductions in Hb were 0.12 (0.95) g/dl and 0.00 (0.55) g/dl for the ACT alone and ACT+SLD-PQ arms, respectively. The mean Hb concentration gradually recovered and was close to baseline on day 28. The Hb level in the ACT alone group recovered by 0.25 (0.81) g/dl and 0.35 (0.66) g/dl for the ACT+SLD-PQ arm at the same follow-up day. The Hb level in the ACT alone group recovered by 0.35 (0.81) on day 21 and by 0.41 (0.63) g/dl on the same follow-up day for the ACT+SLD-PQ arm. The Hb recovery rates were 0.42 (0.86) and 0.45 (0.59) g/dl on day 28 in the ACT alone and ACT+SLD-PQ arms, respectively. The overall pattern showed an initial rapid drop in Hb levels followed by a lengthy recovery period; however, the difference between the two arms was not statistically significant (*p* = 0.157) (Table 3). On day 7, the mean Hb reduction was comparable between the groups (Table 3); for ACT alone and ACT+SLD-PQ, the difference (ACT + SLD-PQ-ACT alone) was 0.20 g/dL (95% CI −0.08–0.47; *P* = 0.157) after adjusting for baseline.

### Hb reduction by G6PD phenotype and treatment arm

To address concerns about hemolysis associated with SLD-PQ use in G6PDd individuals, Hb concentrations were assessed at enrollment and throughout follow-up. Table 4 shows that the mean Hb concentration decreased with the G6PD phenotype. When all the G6PDd patients were phenotypically combined, compared with those in G6PDn patients, the mean Hb reduction was −0.24g/dl (*P* = 0.359). Overall, the baseline mean (±SD) Hb concentrations were similar between G6PDd patients and G6PDn patients [12.77 (1.01) g/dL vs. 12.70 (0.64) g/dL] and during each follow-up period [D7 12.14 (0.66) g/dL vs. 12.38 (1.05) g/dL; D14 12.45 (0.75) g/dL vs. 12.70 (0.87) g/dL; D21 12.54 (0.78) g/dl vs. 12.77 (0.88) g/dL; D28 12.62 (0.79) g/dl].

### Mean Hb concentration reduction by G6PD phenotype

A paired t test model showed a negative association between D7-D0, D14-D0, D21-D0, and D28-D0 decreases in the G6PDd and G6PDn treatment groups, but G6PDn, in comparison to G6PDd, was linked to a positive change in D28-D0 Hb, with a mean increase of 0.05 g/dl (95% CI: −0.14, *P* = 0.316). The decrease in the mean Hb concentration after D 7 was greater in the G6PDd group than in the G6PDn group, but the difference was not significant (*P* =0.359): −0.56 g/dl vs. −0.39 g/dL, respectively; ΔHb concentration = −0.24 (−0.75, 0.27) g/dL. Table 4 shows the reduction in the mean Hb concentration according to the G6PD phenotype. On day 14, the mean decreases in Hb in the G6PDn and G6PDd groups were nearly equal to their values from the previous day, which were −0.07 g/dl and −0.25 g/dl, respectively. On day 21, however, there was no mean Hb decrease in G6PDn individuals, but there was a mean Hb decrease of −0.16 g/dl in G6PDd individuals. On day 28, the mean Hb reduction in G6PDd patients was −0.102 g/dl; however, the mean increase in Hb in G6PDn patients was 0.05 g/dl. This difference was not significant (*P*= 0.337).

The mean changes in Hb levels on day 7 were slightly greater in G6PDd (from 12.70 g/dl to 12.14 g/dl; Δ mean = 0.56 g/dl) individuals than in G6PDn (from 12.77 g/dl to 12.38 g/dl; Δ mean = 0.39g/dl) individuals. were also observed.

**Figure 4:**
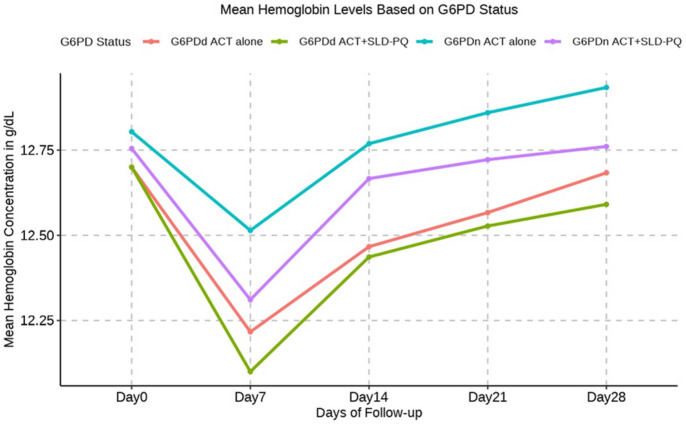
Mean hemoglobin concentrationsin G6PDdand G6PDn study participants who received ACT+SLD-PQor ACT alone.

In comparison to those with G6PDn ACT alone, both the ACT alone and the ACT+ SLD-PQ G6PDd cohorts experienced a lower mean Hb concentration, with mean changes ranging from 0.45 g/dl [95% CI: −0.486 to 0.079] to 0.54 g/dl [95% CI: −1.067 to 0.238] (Table 5 and Figure 4). In addition, on day 7 following ACT+SLD-PQ treatment, the Hb concentration in these G6PDd participants ranged from 12.70 to 12.10 g/dL, and that in the ACT-alone G6PDd participants ranged from 12.70 to 12.22. The distribution of patients between treatment arms was unaffected by sex or phenotypic G6PD phenotype. Although the mean changes in Hb in these groups were greater than those in the G6PDn ACT treatment group, a significant difference in Hb levels was not detected on day 7 posttreatment for G6PDd ACT+SLD-PQ vs. G6PDn ACT alone (*P* = 0.109, *P* = 0.304; Table 5).

However, the absolute mean reduction in Hb levels on day 7 was not significantly lower in either G6PDd (−0.54 g/dl 95% CI: −1.19, 0.12; *P* = 0.109) or G6PDn (−0.20 g/dl 95% CI: −0.48, 0.08; *P* = 0.154) ACT+SLD-PQ individuals (Table 5). There were consistently lower Hb concentrations in G6PDd participants treated with ACT+SLD-PQ than in G6PDn participants, although these differences were not clinically significant.

In the weeks following treatment with ACT alone or ACT + SLD-PQ, the Hb concentration relative to the total concentration gradually decreased; however, it initially decreased in both treatment groups and remained lower until day 28 (Figure 4). On day 14, the mean change in Hb concentration among G6PDd individuals receiving ACT+SLD-PQ was −0.45 (95% CI, −1.01 −0.10, *P*= 0.108), whereas their G6PDn counterparts experienced only a minor change of −0.10 (95% CI, −0.34 −0.13; Table 6). There was no significant difference in the mean Hb concentration between G6PDd ACT-alone patients and G6PDn ACT-alone patients (*P* = 0.234).

In this study, we first reported changes in the mean Hb concentration relative to the baseline value (Figure 3) and the mean changes in the Hb concentration relative to the G6PD status (Figure 4). Mean hemoglobin concentrations, expressed as an absolute or relative change, decreased in the first week following therapy in all ACT+SLD-PQ-treated groups and recovered to baseline levels at follow-up (Figure 4). Similarly, one week after the administration of ACT+SLD-PQ, G6PDd individuals showed a decrease in their hemoglobin concentration compared to that at baseline (Figure 4). The mean Hb levels did not significantly differ between the patients treated with ACT alone and those treated with ACT+SLD-PQ on day 7 (coefficient, −0.45; 95% CI, −1.01, 0.10; *P* = 0.108).

### Severe adverse events in the treatment groups

In our study, abdominal pain, appetite loss, fatigue, and nausea were the major AEs, followed by skin rash, cough, headache, diarrhea, and vomiting. Out of the 249 study participants who completed the follow-up, 94 AEs were recorded—51 (30.7%) from the ACT+SLD-PQ cohort and 43 (51.8%) from the ACT alone group (Table 5). All of the reported AEs were rated as mild. The difference in the incidence of adverse events (AEs) between the treatment groups was not significant (Table 7). There were no SAEs in either treatment group. None of the participants required blood transfusions. In addition, during the study follow-up, every adverse event (AE) resolved, and none of the adverse events stopped participating.

## Discussion

This observational cohort study assessed the safety of combining SLD-PQ (0.25 mg base/kg PQ) with ACT in G6PDd patients to treat uncomplicated *P. falciparum* malaria. Reductions in the mean Hb concentration were considered a surrogate for hemolysis. Various studies examining the relationship between G6PDd and posttreatment hemolysis induced by oxidant antimalarial drugs have used Hb concentration (mean decrease) as the primary outcome measure because changes in Hb levels are objectively measurable [19, 21–23, 48, 49]. In the present study, a dramatic decrease in the mean Hb concentration was observed one week after initiating treatment, and the Hb concentration returned to normal within 28 days. However, there was no significant difference between the ACT alone and ACT+SLD-PQ treatment groups (*P* = 0.157). A similar finding was documented in a previous study from Burkina Faso [25]. Hemolysis of parasitized red blood cells may be the main reason for this reduction in Hb levels at the beginning of malaria treatment, regardless of the patient’s G6PD status[50–52]. After adjusting for age, parasite density, temperature, and sex, a decrease in Hb was observed in the G6PDd patients compared to the G6PDn patients; however, this difference was not significant (*P* = 0.110). This mean reduction in Hb levels was generally insignificant in G6PDd and G6PDn ACT+SLD-PQ individuals.

Mean changes in Hb concentration provided a broad comparison between groups; maximal reductions might more accurately represent extreme hemolysis[53]. The baseline Hb concentration was taken into account when comparing the results, and the maximum reductions in Hb concentration (the largest reduction in Hb concentration compared to baseline at any time point during follow-up) were greater in both the ACT alone and ACT+SLD-PQ groups on day 7 posttreatment, with a relative mean difference of −0.20 g/dL (95% CI, −0.08, 0.47; *P* = 0.157).

There were consistently lower Hb concentrations in G6PDd patients treated with ACT+SLD-PQ than in G6PDn patients, although these differences were not statistically significant. The mean Hb levels in both treatment groups changed, and after day 7, there were indeed significant changes in the Hb levels (*P* < 0.01). The study indicated that ACT+SLD-PQ induced a brief reduction in Hb levels in G6PDd individuals. According to the study, Hb levels in G6PDd patients temporarily decreased after they were treated with ACT+SLD-PQ. Another study [54] that assessed the safety of this SLD-PQ as a transmission blocking therapy showed comparable decreases in Hb levels.

The findings of this study were consistent with those of previous studies indicating low safety concerns associated with SLD-PQ in G6PDd malaria patients[19, 22, 27, 55]. Similarly, a recent systematic review[56] reported that the hemolytic effects of SLD-PQ (0.1 and 0.25 mg/kg) were less likely in individuals who received G6PDd than in those who received a previous dose of 0.75 mg/kg. Overall, these findings imply that reduced G6PD enzyme activity may not lead to a considerable reduction in Hb levels following day 7 after ACT+SLD-PQ therapy. In contrast, a pilot study evaluating the safety of SLD-PQ in Swaziland reported greater reductions (0.6 g/dL) in the mean Hb concentration after one week of SLD-PQ treatment than on day 0[21]. Similarly, a study from Senegal showed that G6PDd patients had significantly lower Hb levels than G6PDn patients on day 7 posttreatment[20]. Additionally, SLD-PQ treatment was linked to a temporary drop in Hb levels in G6PDd individuals[55].

In Africa, G6PDd constitutes up to 37.5% of the population[16], and the use of PQ has been widely restricted due to safety concerns. The present study revealed that the SLD-PQ was safe regardless of the G6PD status.

One limitation of this study was the small number of G6PD-deficient study participants, and an unequal number of study participants were followed from the two study arms. Furthermore, investigations using large population-based cohorts are recommended to strengthen the findings of this study on the safety of the SLD-PQ in G6PDd patients.

## Conclusion

The findings of this study indicate that the use of SLD-PQ in combination with ACT is safe for uncomplicated *P. falciparum* malaria regardless of the patient’s G6PD status. Therefore, it is important to note the broader use of SLD-PQ without prior G6PD testing for the treatment and subsequent elimination of *P. falciparum* malaria.

## Figures and Tables

**Table 1:** Baseline sociodemographic, dinical, parasitological profiles and prognostic profiles of the study participants (n = 249) by treatment group in Arjo-Didessa, southern Ethiopia; 2019–2022

	Treatment arms		
Characteristics			*P*-value
	
	ACT alone	ACT+SLD-PQ	
	83 (33.33%)	166 (66.67%)	

Gender (% male no. of males/total no. of individuals)	65.06(54/83)	64.46(107/166)	0.9257

Age(years), median (IQR)	20(13)	20 (15)	0.9276

Age groups			
<15 years, no. (%)	14(16.87)	37(22.29)	0.405
>15 years, no. (%)	69(83.13)	129(77.71)	

Axillary temperature (°C), mean (SD)	38.13(0.54)	37.98(.63)	0.0759

Fever (≥37.5°c) at present, no. (%)	63(75.90)	119(71.69)	

Asexual parasite density/microliter, mean (SD)	13815.67(19655.76)	17430.92(30420.42)	0.3259

G6PD activity**, no. (%)			
-Normal	77(92.77)	155(93.37)	
-Deficient	6(7.23)	11(6.63)	

Hb concentration at enrollment (g/dl), mean (SD)	12.80(1.18)	12.75(0.89)	0.736

G6PDd Hb(g/dl), mean (SD)	12.70(0.88)	12.7(0.53)	1.0000
G6PDn Hb(g/dl), mean (SD)	12.80(1.21)	12.76(.91)	0.730

Abbreviations: IQR, interquartile range; °C, degree Celsius, number [Note: means and standard deviation are presented for temperature and hemoglobin; age is presented as median and interquartile range].

** as determined by a POCT analyzer indicating G6PD enzyme activity (IU/g Hb)].

**Table 2.** Mean change and mean hemoglobin concentration at baseline and day 7 in the treatment group in Arjo-Didessa, southern Ethiopia; 2019–2022

Treatment groups	N	Mean (Baseline)	Mean (on day 7)	Mean Diff	t -value	*P* value

ACT alone	83	12.79639	12.49277	0.3036145	3.6	0.0005*
ACT+ SLD-PQ	166	12.7512	12.29699	0.454216	13.75	0.0000*

Key: ACT, artemisinin combination therapy; PQ, primaquine. [Note: n, number of observations; mean baseline, mean of baseline hemoglobin concentration in g/dl; mean on day 7, mean of day 7 follow-up time hemoglobin concentration in g/dl; Diff; mean difference (95% CI) estimated using a paired Student t test comparing the mean concentration of hemoglobin on day 7 in each group].

**Table 3.** The mean Hb concentration in each treatment arm and estimation of the mean Hb change and differences from the baseline at each follow-up period using the mixed model in Arjo-Didessa, southern Ethiopia; 2019–2022

	Mean Hb Concentration (SD)		Mean Hb chanae (SD)		mean Hb change difference(95% CI)	
Day	ACT alone	ACT+SLD-PQ	ACT alone	ACT+SLD-PQ	(ACT+SLD-PQ)- (ACT alone)	*P* value
**0**	12.80(1.18)	12.75(0.89)	*	*	*	*
**7**	12.49(1.16)	12.30(0.95)	0.30(0.77)	0.45(0.43)	0.20(−0.08, 0.47)	0.157
**14**	12.75(1.01)	12.65(0.78)	0.05(0.79)	0.10(0.68)	0.10(−0.13, 0.33)	0.412
**21**	12.84(.97)	12.71(0.82)	−0.04(0.90)	0.04(0.61)	0.13(−0.10, 0.36)	0.270
**28**	12.92(.93)	12.75(0.85)	−0.12(0.95)	0.00(0.55)	0.17(−0.07, 0.40)	0.161

Key: ACT, artemisinin combination therapy; 95% CI, confidence interval; Hb, hemoglobin; SLD-PQ, single low-dose primaquine. The mean absolute change was defined as the mean Hb level on the day of follow-up minus the mean Hb level on day 0.

* baseline mean Hb only.

**Table 4.** The mean hemoglobin concentration and range of change from baseline for patients with and without G6PD deficiency on day 7 after treatment.

Group	No. participants	Mean Hb	*P* value
**G6PDd**	17	12.141	
**G6PDn**	232	12.379	0.359

Keywords: G6PDdd, glucose-6-phosphate dehydrogenase deficiency; G6PDn, normal glucose-6-phosphate dehydrogenase.

**Table 5.** Results of multiple linear regression showing the mean Hb concentration and range of change from baseline for patients with and without G6PD deficiency on day 7.

Day 7	Coef.	t value	[95% CI]	p- value

Sex: base male	0	.	.	.
Female	−0.23	−1.64	−0.50–0.05	0.102
Age	0.01	1.71	−0.00–0.02	0.089
**G6PD status**				
G6PDd ACT alone	−0.45	−1.03	−1.31–0.41	0.304
G6PDn ACT+SLD-PQ	−0.20	−1.43	−0.48–0.08	0.154
G6PDd ACT+SLD-PQ	−0.54	−1.61	−1.19–0.12	0.109

Keys: G6PD-d, glucose-6-phosphate dehydrogenase deficiency; G6PD-n, normal glucose-6-phosphate dehydrogenase. [Note: Coef is the mean Hb coefficient of variation estimated using a multiple linear regression model comparing the mean reduction in Hb concentration on day 7 in each group.]

**Table 6.** The mean hemoglobin concentration and range of change from baseline to day 14 for patients with and without G6PD deficiency.

Day 14	Coef.	t value	[95% CI]	p value

Sex: base male	0	.	.	.
Female	−0.016	−1.40	−0.39 –0.07	0.163
Age	0.012	2.29	0.00 – 0.02	0.023
**G6PD status**				
G6PDd ACT alone	−0.44	−1.19	−1.17– 0.29	0.234
G6PDn ACT+SLD-PQ	−0.10	−0.85	−0.34– 0.13	0.397
G6PDd ACT+SLD-PQ	−0.45	−1.61	−1.01 – 0.10	0.108

Keywords: G6PD-d, glucose-6-phosphate dehydrogenase deficiency; G6PD-n, normal glucose-6-phosphate dehydrogenase. [Note: Coef, mean Hb coefficient of variation estimated using a multiple linear regression model comparing the mean reduction in Hb concentration on day 14 in each group.]

**Table 7.** Adverse Events (AEs) Among Study Participants by G6PD Status and Treatment Group, Arjo Didessa, Ethiopia

	Treatment arms				
Adverse events	ACT only		ACT+SLD-PQ		*p*- value
	G6PDn n (%)	G6PDd n (%)	G6PDn n (%)	G6PDd n (%)	
Head ache	4(11.43%)	0	1(2.38%)	1(11.11%)	0.097
Nausea	6(17.14%)	1(12.5%)	4(9.52%)	1(11.11%)	0.062
Vomiting	1(2.86%)	0	1(2.38%)	1(11.11%)	0.705
Abdominal pain	5(17.14%)	2(25%)	12(28.57%)	2(22.22%)	0.460
loss of appetite	5(14.29%)	2(25%)	9(21.43%)	1(11.11%)	0.321
Fatigue	6(17.14%)	1(12.5%)	5(11.91%)	1(11.11%)	0.098
Skin rashes	2(5.71%)	1(12.5%)	5(11.91%)	1(11.11%)	0.626
Cough	4(11.43)	1(12.5%)	3(7.14%)	0	0.084
Diarrhea	1(2.86%)	0	2(4.76%)	1(11.11%)	0.593
Total	35(100%)	8(100%)	42(100%)	9(100%)	

## Abbreviations

ACT: Artemisinin-based combination therapy
AMM: adjusted male median (AMM)
CI: Confidence interval
FMoH: Federal Ministry of Health
G6PD: Glucose-6-phosphate dehydrogenase
G6PDd: Glucose-6-phosphate dehydrogenase deficient
Hb: hemoglobin
ICEMR: International Center of Excellence for Malaria Research
IQR: Interquartile Range
NIH: National Institute of Health
*P. falciparum*: *Plasmodium falciparum*
SD: standard deviation
SLD-PQ: single low-dose (0.25mg/kg) primaquine
WHO: World Health Organization
